# Health actions and occupational vocal conservation: how are they being implemented?

**DOI:** 10.47626/1679-4435-2022-961

**Published:** 2024-08-05

**Authors:** Vanessa Maria da Silva, Maria Luiza Lopes Timóteo de Lima, Natália Melina Mendonça Guimarães

**Affiliations:** 1 Saúde Ocupacional, Caixa de Assistência dos Funcionários do Banco do Brasil, Recife, PE, Brazil; 2 Departamento de Fonoaudiologia, Universidade Federal de Pernambuco, Recife, PE, Brazil; 3 Pós-graduação em Fonoaudiologia do Trabalho, Instituto de Desenvolvimento Educacional, Recife, PE, Brazil

**Keywords:** occupational health, voice, occupational health services, saúde do trabalhador, voz, serviços de saúde do trabalhador

## Abstract

The actions in Occupational Speech Therapy are becoming more evident with the emergence of new experiences and interventions that aim to intercept the disease process, emphasizing the prevention and protection of vocal health in the occupational context. This work aims to analyze how health and occupational vocal conservation actions are being implemented. This is an integrative literature review, whose survey was carried out from June to October 2020, in the Capes Periódicos platform, using the descriptors “worker’s health” and voice. After applying the defined exclusion and inclusion criteria and subtraction of repeated publications, a total of 16 articles were selected. The teacher’s voice has been a priority object in Brazilian speech therapy research in recent years. The results presented show the importance of health promotion actions that aim at the well-being of workers as a whole, in an integral and multidisciplinary way. Occupational vocal health and conservation actions encompass activities related to vocal health surveillance and risk conditions for the development of voice disorders, vocal health education, direct vocal interventions, voice assessment, laryngological assessment, referrals and assessment of workers’ perception of the proposed actions.

## Abstract (pt)

As ações em Fonoaudiologia do Trabalho vêm se tornando mais evidentes com o surgimento de novas experiências e intervenções que visam interceptar o processo da doença, enfatizando a prevenção e a proteção da saúde vocal no contexto ocupacional. Este trabalho visa analisar como estão sendo implementadas as ações de saúde e conservação vocal ocupacional. Tratou-se de uma revisão bibliográfica integrativa, cujo levantamento foi realizado no período de junho a outubro de 2020 na plataforma Periódicos CAPES, utilizando-se os descritores “saúde do trabalhador” e “voz”. Após a aplicação dos critérios de exclusão e inclusão definidos e subtração das publicações repetidas, foi selecionado um total de 16 artigos. A voz de professores vem sendo objeto priorizado nas pesquisas fonoaudiológicas brasileiras nos últimos anos. Os resultados apresentados elencam a importância de ações de promoção da saúde, que objetivem o bem-estar dos trabalhadores como um todo, de forma integral e multidisciplinar. As ações de saúde e conservação vocal ocupacional englobam atividades relacionadas à vigilância da saúde vocal e de condições de risco para o desenvolvimento dos distúrbios de voz, à educação em saúde vocal, a intervenções vocais diretas, à avaliação da voz e laringológica, a encaminhamentos e à avaliação da percepção dos trabalhadores diante das ações propostas.

## INTRODUCTION

Vocal production occurs through the vibration of the vocal folds, triggered by the passage of expiratory air through the larynx, the sound of which is amplified and filtered by the resonance chamber. This process involves the pharynx, oral cavity, sinuses and nasal cavity. In addition to the phonoarticulatory organs, vocal production involves the central nervous system, as well as emotional aspects.^1^

The voice is of fundamental importance for effective communication, enabling interpersonal relationship and the expression of meanings and individual characteristics of the speaker on a psychological and social level, through the externalization of feelings and thoughts.^2^

. In some professions, the voice is essential for making work viable, being a crucial instrument. The occupational voice is defined as “the form of oral communication used by individuals who depend on it to perform their occupational activity.”^3^ Teachers, singers, actors, religious people, politicians, secretaries, lawyers, prosecutors, judges, health professionals, salespeople, street vendors, community workers, ceremonialists, broadcasters, journalists, teleoperators, among others, use their voice professionally.

Depending on their professional activity, excessive workload, adverse working conditions, biological, emotional, or environmental interference, their voices can become altered and have pathologies.^4^

As a symptom, dysphonia is any alteration that prevents the natural production of the voice, with emotional, social and economic and, above all, professional impacts, as in the case of workers who depend on vocal production and/ or a specific vocal quality for their professional survival, whose effects on vocal quality are quite variable and can range from mild to severe.^5^ Work-related voice disorder (WRVD) is therefore any form of vocal deviation related to professional activity that reduces, compromises, or prevents the performance or communication of the worker, whether or not there is an organic alteration of the larynx.^4^

This high prevalence of voice disorders in the workplace is a sign of collective illness, caused by the wear and tear on the voice under precarious occupational conditions.^6^ Epidemiological studies have been compiled in the WRVD protocol, evidencing the high prevalence of voice disorders, especially in teachers, based on their symptoms, predisposing personal factors and environmental and organizational risks at work.^4^ There have been few epidemiological studies on other voice professionals, which makes it difficult to plan and develop actions aimed at other risk groups.

Occupational safety involves all aspects of health in the workplace. Occupational illnesses, violence, moral, and sexual harassment, work-related accidents and other issues are just some of the topics that should be addressed. Recommendations on vocal hygiene are important in this context, as they include prevention of problems that can affect voice, not just those concerning the vocal apparatus, but also other organs, functions, and conditions that can indirectly affect the vocal tract.^7,8^

In addition to initiatives and studies to include WRVD as an occupational disease, the programs described by occupational health managers have expanded actions in discussion circles in educational institutions to provide courses, training, and primary interventions in the environment, adapting it to the preservation of vocal health.^6^ Speech and hearing therapy is responsible for performing preventive vocal health actions through campaigns, providing advice, courses, and lectures to alert people who use their voices professionally to signs and symptoms of vocal alterations and to provide information on vocal hygiene.^9^

Occupational speech therapy prevention actions have become more evident with the emergence of new work proposals and experiences involving actions to intercept the occupational disease process, emphasizing health prevention and specific protection. Recently, WRVD was included in the list of occupational diseases,^10^ and the Brazil Ministry of Labor and Employment has revised its regulations.^11,12^ This has encouraged the development of programs and actions aimed at vocal health in the occupational context.

Given that speech and hearing therapy has an important role to play at the intersection of education and health, and considering the need to update speech and hearing therapy practice in actions to prevent WRVD, this study aimed to analyze how occupational vocal conservation actions are being implemented.

## METHODS

This is an integrative, qualitative bibliographic review, based on a survey and analysis of academic articles on the subject. Studies of this nature enable the synthesis of knowledge and the applicability of results of studies considered significant to the field.

The bibliographic search was conducted between June and September 2020, on the CAPES Periódicos platform, using the following terms on the Medical Subject Headings: “saúde do trabalhador” and “voz”.

The inclusion criteria were original and complete scientific articles involving professional/occupational voice and published in Portuguese.

The materials that presented content pertinent to the questions proposed in this study were screened and selected for review. The search strategy was built and conducted based on the following question: “What vocal conservation actions are being developed among professionals who use their voices professionally?”.

The Boolean operator used was “AND”, and the pair of descriptors used was “saúde do trabalhador” AND “voz”.

According to the search strategy, the articles found underwent three stages:

- Reading the titles in the electronic database;
- Reading the abstracts of the studies screened in the first stage; and
- Reading the full text to select those to be included in this review.

**Figure 1:**
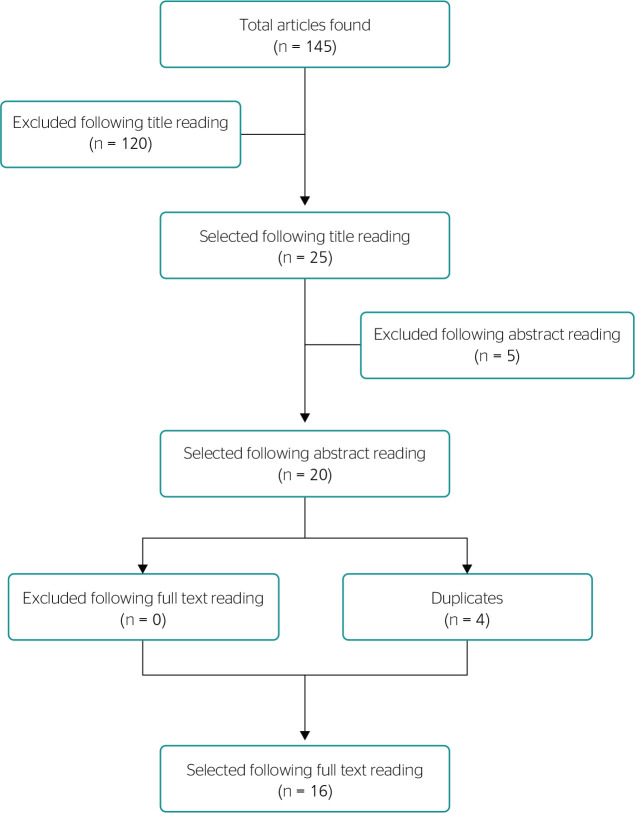
Flowchart of selection criteria for the review.

Figure 1 shows a flowchart illustrating the study selection criteria.

All the articles included met the inclusion criteria defined in this study design. The primary information from each text was collected and entered into a database on an MS Office Excel 2016 spreadsheet. The results were presented using the following variables: author, place of publication, year, journal, study design, sample size, audience, and actions implemented.

The main findings related to occupational vocal health actions were grouped into categories, due to the similarity of content and because they responded to the same objective.

## RESULTS

After cross-referencing, a total of 145 articles were found. After applying the exclusion and inclusion criteria and removing duplicates, 16 articles were selected. Table 1 shows the articles selected.^13-28^

**Table 1:** Studies included in this review

Author	Place of publication	Year	Journal	Study design	Sample size	Audience	Main findings
Santana et al^21^	São Paulo, Brazil	2012	*Jornal da Sociedade Brasileira de Fonoaudiologia*	Literature review	32	Teachers	Identifying the risk factors associated with vocal disorders in teachers, transforming working conditions and guaranteeing the quality of care.
Trigueiro et al.^23^	Brazil	2015	*Revista de Pesquisa Cuidado é Fundamental Online*	Experience report	90	Teachers	Group workshops, held weekly and lasting 2 hours each, involving vocal disorders, their impacts, causes, signs, symptoms, and harmful factors; vocal hygiene guidelines, relaxation/ breathing exercises; articulation exercises and vocal warm-up and cool-down exercises (before and after class).
Almeida et al^24^	Universidade de Fortaleza, Brazil	2012	*Revista Brasileira em Promoção da Saúde*	Qualitative action research	12	Teachers	Situational analysis of the teachers (complaints and symptoms); five theoretical-practical bi-weekly meetings lasting 45 minutes, involving vocal hygiene, body awareness, breath control, stretching and relaxation, posture improvement, and vocal warm-up exercises.
Dragone^16^	São Paulo, Brazil	2011	*Revista Centro de Especialização em Fonoaudiologia Clínica*	Experience report	396	Educators: Teachers, nursery workers, recreationists, educational agents, and managers	Introduction of basic and advanced voice groups depending on the presence of signs and symptoms of vocal disorders, with activities geared to the demands of each group; theoreticalpractical meetings involving vocal behaviors related to teaching practice, information on vocal production and care, training in basic phonatory tasks to increase vocal endurance and reduce strain; application of a protocol for self-perception of the severity of voice problems and voice interference in professional and social activities; perceptual-auditory assessment of vocal quality (GRBASI scale); speech therapy assessment of the voice; referral of altered cases for otorhinolaryngological assessment; guidance on seeking medical care and referral for individual speech therapy were formally provided when necessary.
Pereira et al.^25^	São Paulo, Brazil	2015	*Revista de Saúde Pública*	Parallel-group, single-blinded, randomized clinical trial	31	Teachers	A protocol for self-assessment of the voice was applied; computerized acoustic analysis of the voice was performed; guidance was given to perform vocal warm-up exercises and breathing training once a day, lasting an average of 13 minutes, before the working day; a postintervention questionnaire was applied to assess the participants’ perception after the actions.
Souza et al.^17^	São Paulo, Brazil	2017	*Revista Centro de* *Especialização em* *Fonoaudiologia Clínica*	Single-group, single-blinded intervention study	29	Teachers	Survey of working conditions; vocal assessment before and after the intervention; vocal selfassessment; perceptual-auditory speech assessment; daily SOVT exercises with a commercial straw for 4 weeks, in the morning and evening shifts before starting the work shift.
Luchesi et al.^18^	São Paulo, Brazil	2012	*Revista Centro de* *Especialização em* *Fonoaudiologia Clínica*	Experience report	5	Teachers	Laryngological examination; perceptual-acoustic analysis before and after the interventions; implementation of a vocal improvement program: 12 90-minute weekly meetings covering notions of phonatory anatomy and physiology, vocal health (habits and care), breathing, pneumophonoarticulatory coordination, phonatory strain, articulation, speed and modulation of speech, resonance, vocal projection, verbal and nonverbal expression, vocal warm-up and cool-down.
Luchesi et al.^19^	São Paulo, Brazil	2010	*Revista Centro de* *Especialização em* *Fonoaudiologia Clínica*	Experience report	26	Teachers	A questionnaire to understand vocal and occupational demands, vocal complaints, and suggestions for action; laryngological examination; preventive-therapeutic group intervention, carried out in 12 90-minute weekly meetings, discussing phonatory anatomy and physiology, vocal health (habits and care), breathing, pneumophonoarticulatory coordination, phonatory strain, articulation, speed and modulation of speech, resonance, vocal projection, verbal and nonverbal expression, vocal warm-up and cool-down.
Penteado & Ribas^22^	São Paulo, Brazil	2011	*Jornal da Sociedade* *Brasileira de* *Fonoaudiologia*	Literature review	NA	Teachers	Educational actions in teachers’ vocal health, including vocal behaviors (abuse/bad use) and habits, hygiene/vocal health care; warm-up and cool-down exercises, vocal techniques, anatomy, physiology and vocal production, orofacial motricity, and stomatognathic functions.
Anhaia et al.^26^ Almeida et al.^27^ Luchesi et al.^14^	São Paulo, Brazil São Paulo, Brazil São Paulo, Brazil	2013 2010 2009	*Audiology -* *Communication* *Research* *Arquivos* *Internacionais de* *Otorrinolaringologia Saúde e Sociedade*	Literature review Cross-sectional study Qualitativedescriptive case study	9 328 25	Teachers Teachers Teachers	Review of direct intervention practices, through vocal training, and indirect interventions, through consultancy or education on vocal hygiene and improving the acoustic conditions of the work environment. Drafting and validating a self-assessment questionnaire to measure the prevalence of dysphonic syndrome symptoms and determine the characteristics of the population that may be at risk of developing the disease; it can be used by occupational physicians for epidemiological control of the population, and by otorhinolaryngologists to guide the indication of more complex procedures. Laryngological examination, preventivetherapeutic intervention in a group, speech therapy assessment before and after participation in the group and interviews; suggestions for action from the teacher’s point of view.
Masson et al.^28^	São Paulo, Brazil	2019	*Revista* *Communication* *Disorders, Audiology and Swallowing*	Single-blinded, control group, exploratory quasi- experimental study	18	Teachers	A questionnaire was used to collect sociodemographic data and the worker’s functional situation; perceptual and auditory vocal assessment; acoustic analysis of the voice; self-assessment of the degree of vocal discomfort; vocal warm-up before the class, which lasted 13 minutes with a 30-second break after each series of exercises, involving body, neck and vocal tract stretching, rib cage expansion, phonoarticulatory exercises, air direction, mucosal flexibilization and resonance; vocal cool-down after class, which lasted 7 minutes and involved body and neck stretching, expansion of the pharyngeal cavity, reduction of the fundamental frequency, intensity, and laryngeal strain.
Penteado et al.^15^	São Paulo, Brazil	2009	*Revista Centro de* *Especialização em* *Fonoaudiologia Clínica*	Experience report	20	Receptionists, telephone operators, secretaries, administrative assistants, nurses, social work staff, and health professionals	Group activities, held in seven 75-minute meetings; introduction of the participants to form the group; survey of the subjects’ perceptions of their own voice (vocal image, complaints, needs, and interest in vocal improvement) and demands of professional use of the voice combined with information on aspects of the workplace, conditions and organization; vocal warm-up exercises; survey of professional uses of the voice in work contexts; stimulating the subjects’ attention to the use of the professional voice and to vocal quality and its possible impacts on relationships; vocal assessment of the participants and pointing out possibilities for improvement; approach to the theme of vocal health and improvement of vocal and body expression; reflection on the process experienced, indicating the changes realized.
Brasil et al.^13^	Lousada, Portugal	2020	*Revista Ibérica de Sistemas e* *Tecnologias de Informação*	Applied, methodological study	40	Teachers	An application for a mobile device aimed at promoting vocal health, including tests, tips and guidelines, reports comparing vocal performance, game strategies to motivate use, alerts and reminders to drink water, a tool for capturing environmental noise.
Ribas et al.^20^	São Paulo, Brazil	2014	*Revista Centro de* *Especialização em* *Fonoaudiologia Clínica*	Quasiexperimental study	20	Teachers	Survey of vocal complaints through the application of a protocol; voice experience groups, through three monthly meetings, lasting 45 to 50 minutes each, involving vocal production, conditions, and organization of teaching duties.

SOVT = semi-occluded vocal tract exercises; NA= not applicable; GRBASI scale = G - grade, R - roughness, B - breathiness, A - asthenia, S - strain and I - instability.

All studies except one published in Portugal^13^ were published in Brazil. The earliest studies date back to 2009,^14,15^ and the latest study is from 2020.^13^

Among the articles included, seven were published in the journal Centro de Especialização em Fonoaudiologia Clínica (CEFAC),^15-20^ two were published in the Jornal da Sociedade Brasileira de Fonoaudiologia,^21,22^ and one study was found in each of the following journals: Revista de Pesquisa: Cuidado é Fundamental Online,^23^ Revista Brasileira em Promoção da Saúde,^24^ Revista de Saúde Pública,^25^ Audiology - Communication Research,^26^ Arquivos Internacionais de Otorrinolaringologia,^27^ Revista Saúde e Sociedade,^14^ Revista Communication Disorders, Audiology and Swallowing,^28^ and Revista Ibérica de Sistemas e Tecnologias de Informação.^13^

In terms of sample size, the largest study had 396 participants,^16^ and the smallest study had five participants.^18^ One of the studies omitted the number of participants included in the measures.^22^

The study designs included literature reviews,^21,22,26^ experience reports,^16,18,19,23^ action research,^24^ randomized clinical trials,^25^ intervention studies,^17^ cross-sectional studies,^27^ case studies,^14,15^ exploratory quasi-experimental studies,^20,28^ and applied methodological studies.^13^ Most experience reports validate the experience as a scientific phenomenon, highlighting ways of reading reality and involving ideologies, methodologies, dialogical interactions between subjects, contexts and researchers, and sociopolitical and historical conceptions.^29^

It is particularly worthy noting that the main target audience were teachers. One of the studies involved other educators,^16^ and another involved hospital attendants, administrative assistants, and health professionals.^15^

In general, the actions adopted in these studies included one or more of the following:

- Vocal health monitoring measures;^13-15,17,19-21,27,28^
- Vocal health education;13,15,16,18-20,22-24,26
- Direct vocal interventions;^14-20,22-26,28^ **•** Voice assessment;14,16-20,25,27,28
- Laryngological examination;^14,16,18,19^
- Referrals to an otorhinolaryngologist or individual speech therapy;^16^ and
- Workers’ perception of the proposed actions.^15,25^

## DISCUSSION

The results show that in Brazil, speech therapy research has prioritized the voices of teachers in recent years, and that they are the most studied professional category when compared to workers in other categories. Epidemiological studies with other professional categories also indicate a situation of high prevalence of symptoms and vocal changes. A survey with appliance and furniture salespeople showed the presence of vocal symptoms such as dry mouth and throat (30%), tiredness when speaking (22%), and throat clearing (18%).^30^ Teleoperators currently represent another professional category highly affected with work-related illnesses, including voice disorders.^31^ Community health workers reported voice complaints (42.9%), and the most frequently reported symptoms were hoarseness (33.3%), shortness of breath (32.1%), dry throat (32.1%), and tiredness when speaking (32.1%).^32^

New work contexts and characteristics, such as occupations with high exposure to smoke (working in steakhouses and grills) and to chemicals that can be inhaled (working in beauty salons and cleaning/ general service activities), under extreme temperature conditions (working in meatpacking plants), or with work characteristics that can have an impact on vocal health need to be considered. Additionally, more scientific engagement and research is needed to address the speech therapy practices that have been developed with other voice professionals, considering complaints and symptoms, the organizational context, and specific working conditions.

Occupational health monitoring actions aim to analyze the relationship between health, workplace, and work processes, identifying and/or recognizing occupational risks related to illness processes. It is important to monitor workplaces, to analyze the risk factors associated with voice disorders in voice professionals, and to support the formulation of actions and measures that should be taken to minimize or eradicate these factors.^33^

Preventive actions and vocal health measures should consider the determinants of voice disorders, recognizing that a vocal disorder is not determined simply due to prolonged or excessive use of the voice, but also the existence of other competing factors causing these alterations, especially environmental and organizational factors.^21^

Vocal health monitoring actions discussed in the studies involve assessing work conditions,^17^ knowledge of occupational demands, and the worker’s functional situation,^19,20,28^ addressing these aspects with workers, providing guidance on how to perceive the risks, and accepting suggestions for modifying the conditions observed,^15^ planning actions aimed at working conditions,^14^ and workers monitoring noise in the workplace using an app for mobile devices.^13^

Actions to raise awareness of the risks related to WRVDs should support the search for solutions to reduce or eradicate occupational exposure, so as to preserve voices and promote occupational health. It is crucial that workers are educated, conscious and involved through training, workshops, lectures, campaigns, and guidance. Workers should be familiar with the conditioning factors of WRVD, the signs, and symptoms of vocal disorders and, above all, their role in preventing these symptoms.^34^

Education in vocal health is increasingly based on the principles of andragogy, which is a teaching/ learning method consisting of experiences, previous knowledge, and awareness of the adult individual, who is the protagonist of the proposed changes.^35^

The group workshop “The teacher’s voice: an instrument to be cared for” enabled teachers to assimilate the information essential for health care, enhancing changes in their behavior by showing them how caring for their voice can prevent future problems and minimize current ones. The 2-hour weekly meetings provided guidance on vocal disorders, their impact, causes, signs and symptoms, harmful factors, and vocal hygiene.^23^

A second study held 45-minute fortnightly meetings on vocal hygiene and direct interventions involving body awareness, breathing control, stretching and relaxation, posture improvement, and vocal warm-up exercises.^24^

Dragone^16^ studied basic and advanced groups to develop targeted actions, depending on vocal signs and symptoms in workers and their specific needs. The theoretical-practical meetings focused on teaching-related vocal behaviors, information on vocal production and care.

Some studies have demonstrated the importance of a vocal enhancement programs with 12 90-minute weekly meetings, addressing concepts of phonatory anatomy and physiology, vocal health (habits and care), breathing, pneumophonoarticulatory coordination, phonatory tension, articulation, speed, and modulation of speech, resonance, vocal projection, verbal and nonverbal expression.^18-20^

These educational sessions dealt with teachers’ vocal health, including vocal behavior (abuse/bad use) and habits, hygiene and vocal health care, warming up and cooling down, exercises, vocal techniques, anatomy, physiology and vocal production, orofacial motricity, and stomatognathic functions.^22^

Penteado et al.^15^ organized group activities in seven 75-minute meetings designed to raise workers’ awareness of the use of the professional voice, vocal quality, along with potential impacts on interpersonal and work relationships. The content covered a survey on workers’ perceptions of their own voice (vocal image, complaints, needs, and interest in vocal improvement), an analysis of the demands of professional voice use combined with information on aspects of the environment, working conditions, and organization, as well as a survey of the professional uses of the voice in work contexts.

Ribas et al.^20^ conducted voice experience groups in three monthly meetings, each lasting 45 to 50 minutes, involving vocal production and the conditions and organization of teaching duties.

VoiceGuard^®^ is an application which offers tips and guidance on vocal health, with strategies and games to engage and motivate workers to use and adopt healthy vocal habits.^13^

Anhaia et al.^26^ showed the importance of indirect vocal interventions, which help individuals gain awareness of their vocal use and psychological and environmental factors leading to voice disorders, in order to develop strategies to minimize these risk factors. However, their analysis found that indirect vocal interventions combined with direct vocal interventions (instructions on voice techniques) showed more significant results than intervention alone.

Direct vocal interventions involve specific vocal training, guidance, and warm-up and cool-down exercises. Vocal warm-up exercises prepare the phonation system for intense use of the voice through techniques to control respiratory airflow, head and neck mobilization, and flexibility of the extrinsic and intrinsic muscles of the larynx, reducing the elastic and viscous resistance of the vocal folds and encouraging them to stretch. It also improves voice projection, increasing intensity and reducing vocal effort and fatigue. Vocal cool-down exercises aim to gradually restore the voice to a conversational level, stretching the muscles involved, reducing the strain experienced during intense use of the voice and lowering the intensity and fundamental frequency, which are determining factors in vocal overload. The fact that this process is gradual helps to remove lactic acid, which is responsible for the sensation of pain, and should be performed immediately after intense use of the voice.^36^

Most studies included in this review performed vocal training by warming up before and cooling down after work, showing positive results in terms of workers’ perception of vocal comfort and the prevention of occupational vocal disorders.^14-16,18-20,22-26,28^

In one study^17^, semi-occluded vocal tract exercises were performed with a commercial straw every day for 4 weeks, in the morning and evening before teachers started their work shift. The results of this study showed an improvement in vocal quality after 4 weeks of intervention and the teachers reported beneficial effects. The technique can be used in vocal health programs as a protective measure for the voice in populations that are more exposed to vocal disorders.

Some studies have shown that voice assessments are performed through questionnaires or voice selfassessment forms, with perceptual-auditory analysis and computerized acoustic analysis before and after interventions to measure the efficacy of the actions proposed.^14,16-20,25,27,28^ Voice self-assessment forms can help define the characteristics of the population, guiding health actions aimed at workers and supporting clinical control and monitoring of the data collected.^27^ It should be noted that, for the purposes of early diagnosis and monitoring, it is recommended that every candidate or worker who has or will have their voice as a working tool should have their voice assessed before they take their preemployment medical examination, at the time of any occupational medical examination in the absence of a previous voice examination and/or in the presence of vocal complaints.

Laryngological examinations can complement and confirm information from other tests, providing objective data for the diagnosis and monitoring of vocal disorders. Laryngological examinations with otorhinolaryngologists were one of the actions considered to control and measure the efficacy of the vocal training and exercises provided.^14,16,18,19^

In addition to diagnosing and monitoring signs and symptoms, it is important to consider the need to refer cases of vocal disorders that require more specific testing and/or treatment to an otorhinolaryngologist or individual speech therapy.^16^

Another important parameter aimed to complement the assessment and impact of proposed actions is the worker’s perception. Penteado et al.^15^ highlighted the importance of listening to the impressions, comments, criticisms, suggestions, and changes perceived by workers in the face of vocal health actions, considering the impact on their work and quality of life. Teachers’ suggestions for speech therapy actions aimed at vocal health at schools include vocal education for students, a speech therapist at the educational institution, engaging educational and professional organizations, vocal improvement programs that focus on voice care and vocal techniques, and actions aimed at working conditions.^14^ This information can be considered when planning future actions to meet the main demands.

These findings point to the importance of health initiatives, i.e. actions aimed not only at vocal health, to ensure the overall well-being of the professional, in a comprehensive and multidisciplinary manner.

## CONCLUSIONS

Occupational vocal health and conservation actions encompass activities related to vocal health monitoring and risk conditions associated with WRVD development, vocal health education, direct vocal interventions, voice assessment, laryngological examination, referrals, and workers’ perception of the proposed actions.

For comprehensive planning and action delivery, it is important to consider the changing contexts and characteristics of work, and to encourage further studies and surveys that address speech therapy practices developed with other voice professionals, considering their complaints and symptoms, the organizational context, and their specific working conditions.
